# The Role of Plasticity in Replay: Stability Through Anti‐Hebbian Rules

**DOI:** 10.1002/hipo.70089

**Published:** 2026-04-11

**Authors:** Lior Baron, Kamran Diba, Asohan Amarasingham

**Affiliations:** ^1^ Department of Computer Science CUNY Graduate Center New York New York USA; ^2^ Department of Anesthesiology University of Michigan Medical School Ann Arbor Michigan USA; ^3^ Department of Mathematics The City College of New York New York New York USA; ^4^ Department of Biology CUNY Graduate Center New York New York USA

**Keywords:** CA3 recurrent network, hippocampal replay, memory consolidation, synaptic plasticity

## Abstract

Hippocampal replay is now considered to be a cornerstone of memory consolidation, yet the synaptic plasticity rules governing its dynamics remain elusive. Under the standard asymmetric Hebbian spike‐timing dependent plasticity (STDP) model, the same spike patterns that promote activity propagation along one direction of sequential activation undermine propagation in the reverse direction, compromising “bidirectional” replay. On the other hand, symmetric potentiation rules, as recently proposed for region CA3, risk corrupting the memory trace by saturating synaptic weights. Using Ecker et al.'s recurrent network model of place cells that spontaneously generate replays during ripples, we systematically investigated how different STDP plasticity rules modulate offline replays. We developed a classification framework to study the mechanisms relating different STDP kernels to key replay characteristics, including directionality, speed, and stability. Our results confirmed that symmetric potentiation rules during offline states saturate synapses, inducing rigid attractors that corrupt the memory trace, and that an asymmetric Hebbian STDP kernel induces strong biases in the directionality of replay, leading to rapid replay acceleration and replay degradation. Notably, we found that an asymmetric anti‐Hebbian STDP kernel preserves replay bi‐directionality and stabilizes replay speed. We further identified the negative timing component of the STDP rule as the primary driver of replay speed: potentiation causes deceleration, while depression causes acceleration. These findings provide a mechanistic explanation for empirically observed replay deceleration and suggest a role for anti‐Hebbian synaptic depression in stabilizing replay dynamics.

## 1 Introduction

During brief pauses in behavior and during periods of sleep, often referred to as “offline” periods, hippocampal neurons can rapidly reinstate the firing patterns observed during “online” experience, when animals are learning and exploring (Pfeiffer 2020). These replays unfurl in time‐compressed spike‐train sequences during brief network‐wide population events that reverberate throughout the hippocampal circuit (Chrobak and Buzsaki 1996) and synchronize with other brain circuits (Logothetis et al. 2012) in a “neocortical‐hippocampal” dialogue that has been implicated in both memory consolidation and navigational decision‐making (Rothschild 2019; Ólafsdóttir et al. 2018). This tight relationship between firing during online periods and subsequent offline periods suggests a neuronal mechanism for rapid learning and might serve as a model for studying memory formation and transformation in the brain (Buzsáki 2015). Nevertheless, the synaptic rules underlying the production and dynamics of hippocampal replay remain largely unknown.

Remarkably, the same hippocampal neurons participate in replays that are forward, reflecting their firing order during experience (e.g., for navigation on linear tracks) (Diba and Buzsáki 2007) and in reverse (i.e., reversing the order of experience) (Foster and Wilson 2006). Generating such bidirectional sequences in a network presents a challenge to experience‐dependent Hebbian plasticity rules (Bi and Poo 1998; Levy and Steward 1983), in which synapses are strengthened when presynaptic spiking precedes postsynaptic spiking and weakened when postsynaptic spiking precedes presynaptic spiking. Although the timescale of such plasticity rules is understood to match the timescale of replay, this challenge arises because plastic Hebbian changes that promote sequential activation in one direction would obstruct the propensity for activation in the reverse direction.

**FIGURE 1:**
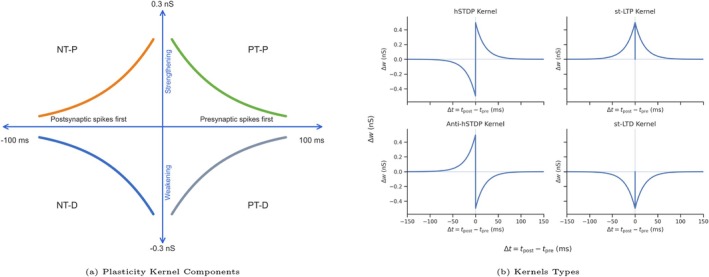
(a) Plasticity Kernel Component (PKC) classification. X‐axis: Spike timing between pre‐ and postsynaptic PCs, defined as (post—pre spike time) in milliseconds—Positive Timing (PT) when x > 0 and Negative Timing (NT) when x < 0. Y‐axis: Change in synaptic conductance (nS); Long Term Potentiation (LTP) for values > 0, Long Term Depression (LTD) for values < 0. PKC labels: NT‐D, depression for negative timing; NT‐P, potentiation for negative timing; PT‐D, depression for positive timing; PT‐P, potentiation for positive timing. (b) Kernel Types: hSTDP: Co‐activation of PT‐P & NT‐D; symmetric timing LTP (st‐LTP): Co‐activation of PT‐P & NT‐P; anti‐hSTDP: Co‐activation of PT‐D & NT‐P; symmetric‐timing LTD (st‐LTD): Co‐activation of PT‐D & NT‐D.

Recent studies have addressed these challenges by either hard‐wiring strong reverse connections into the network (Molter et al. 2007) or, alternately, by adopting non‐Hebbian plasticity rules that incorporate symmetric‐timing long term potentiation (st‐LTP); (Haga and Fukai 2018; Ecker et al. 2022). These latter studies enforced potentiation both when spike timing differences (“lags”) between pre‐ and postsynaptic spikes are positive (with presynaptic spikes preceding postsynaptic spikes; “PT‐P” in Figure 1a) and when such spike timing differences are negative (postpreceding pre; “NT‐P” in Figure 1a). By simultaneously strengthening both forward connections (those compatible with the experienced sequence of activity) and reverse connections (those compatible with the reversed sequence of activity) after the ordered firing of place cells (PCs) during experience, the networks could then produce replays in both directions.

Notably, the CA3 model of Ecker et al. (2022) was particularly impressive in making publicly accessible a biologically plausible network of excitatory principal cells (PCs) and inhibitory basket cells (BCs) that could spontaneously generate sharp‐wave ripple (SWR) oscillations following learning during and in which the recurrently connected PC network displayed both forward and reverse replays. In their study, Ecker et al. (2022) turned off plasticity during the off‐line state in order to focus their examination on the effects of online learning. However, in our study, we aimed to gain a better understanding of the role played by plasticity rules during the off‐line state replay in producing different features of hippocampal replay dynamics. In our implementation of the Ecker et al. (2022) model, we developed a modular plasticity evaluation framework that categorized the selective activation of different components of the STDP kernel during the offline simulation phase. This framework parameterized synaptic plasticity rules based on the relative temporal order of presynaptic and postsynaptic PCs spike timing.

The plasticity implemented by Ecker et al. (2022) involved only synaptic potentiation, that is, via co‐activation of both PT‐P & NT‐P (1b—Top right). We hypothesized that continued application of this synaptic plasticity rule would lead to eventual saturation of synaptic connections and network failure that could not support the original memory trace. By performing simulations with plasticity left on, we found that, as expected, potentiation that was unbalanced by depression ultimately led to unstable network dynamics, whereas depression alone completely erased the memory trace. We then explored plasticity rules that contained both potentiation and depression kernel components (Figure 1b). Remarkably, when potentiation and depression were balanced as in standard Hebbian asymmetric STDP, we found that replays became increasingly faster and biased over time, unlike what is reported in vivo (Berners‐Lee et al. 2022). In contrast, a noncanonical anti‐Hebbian STDP slowed down the speed of replays, consistent with recent experimental observations (Berners‐Lee et al. 2022), but also preserved their bidirectionality without saturation or elimination.

## 2 Methods

### 2.1 Model Overview

We adopted the CA3 network model of Ecker et al. (2022), which is implemented in Python using the Brian2 package (Stimberg et al. 2019). We performed multiple simulations under different parameters as described below. Each simulation consisted of two phases: an *online* *learning phase (Phase 1)* and an *offline replay phase (Phase 2)*.

#### 2.1.1 Phase 1 Online Learning: Point Process Model for Learning

The purpose of the online learning phase is to generate the recurrent PCs (PC‐to‐PC) weight matrix used later to simulate spiking activity in Phase 2. In contrast to other models (e.g., Ramirez‐Villegas et al. 2018, Pang and Fairhall 2019), which impose synaptic weights according to predefined network topology rules, we wanted to adopt a more directly biologically motivated approach by imposing a spiking pattern on the network and calculating the resulting synaptic weights based on the applied plasticity rule.

Therefore, we followed Ecker et al. (2022) by imposing a spiking pattern that simulated the CA3 spiking activity of a rodent traveling in one direction along a 3‐m linear track. The model architecture consisted of a network of 8000 pyramidal cells (PCs) with recurrent connections, where each pair of PCs was independently connected with 10% probability. In each simulation, 50% of the neurons were randomly designated as PCs. Each selected PC was assigned a spatial tuning curve with a Gaussian profile (standard deviation = 7.5 cm). Place fields were generated with centers randomly distributed along the track, and assigned to neurons so that place‐field centers increased monotonically with PC ID.

As in Ecker et al. (2022), spike trains for non‐PCs were modeled as independent homogeneous Poisson processes with a constant mean firing rate of λ=0.1 Hz, while those for PCs were modeled as conditionally independent point processes, firing at peak frequencies of 20 Hz, and with conditional intensity functions $\lambda_{i}\left(t;H_{t}\right)$ (Equation 1) expressing spatial preferences with Gaussian tuning curves, as well as theta‐modulation. ($H_{t}$ here signifies spiking history up to time t.) The Gaussian standard deviation term (σ) was set to cover 10% of the 3‐m track. Finally, spikes occurring within 5 ms of a previous spike were discarded to enforce a refractory period.

As in Ecker et al. (2022), the simulated animal's speed was set to 32.5 cm/s. Each traversal of the linear track took approximately 9.2 s. After reaching the end, position was instantaneously returned to the start of the track. The generated spike trains spanned 400 s, or approximately 43 repetitions of the linear track.

The conditional intensity function of PC i is specified via:

(1) $$\begin{array}{c} \lambda_{i}\left(t;H_{t}\right)=\lambda_{i}\left(t,t-s_{i}^{*}\left(t\right)\right)=\lambda_{\max}\times \tau_{i}\left(x\left(t\right)\right)\\ \;\times \cos\left(2\pi f_{\theta }t+\frac{\pi }{l^{\mathrm{PF}}}\left(x\left(t\right)-s_{i}^{\mathrm{PF}}\right)\right)\times \;1\left\{t-s_{i}^{*}\left(t\right)>t_{\mathrm{ref}}\right\} \end{array}$$

(2) $$\tau_{i}\left(x\right)=\exp\left(-\frac{{\left(x-m_{i}^{\mathrm{PF}}\right)}^{2}}{2\sigma^{2}}\right)$$

where t is time, $s_{i}^{*}\left(t\right)$ is the last spike of the i'th neuron before time t, $1\left\{\cdot \right\}$ is the indicator function, $\tau_{i}\left(x\right)$ is the spatial tuning curve of the i'th neuron, σ=7.5cm is the standard deviation of the Gaussian tuning curve, $x\left(t\right)$ is the position of the animal, $m_{i}^{\mathrm{PF}}$ is the center of the place field, $l^{\mathrm{PF}}=0.3\;m\left(=4\sigma \right)$, and $s_{i}^{\mathrm{PF}}\left(\;=m_{i}^{\mathrm{PF}}-2\sigma \right)$ are the length and start of the given place field, respectively, and $\lambda_{\max}=20\;\mathrm{Hz}$ is the maximum in‐field firing rate, $f_{\theta }$ is the background 7 Hz theta activity. $t-s_{i}^{*}\left(t\right)$ is the time between two consecutive spikes, $t_{\mathrm{ref}}=5\mathrm{ms}$ is the refractory period.

During the phase 1 simulations, synaptic connection strength was modified based on a symmetric STDP rule:

(3) $$\Delta w=A\cdot \exp\left(\frac{\left|\Delta t\right|}{\tau }\right)$$

where Δw is the change in synaptic weights in nS, $\Delta t=t_{\text{post}}-t_{\mathrm{pre}}$ is the time difference in ms between action potentials, and A describes the weight update in nS, which decayed exponentially with time constants τ in ms, respectively. We used the optimized parameters from Ecker et al. (2022) for our phase 1 learning (τ = 62.5 ms and A = 0.08 nS), which were shown to produce robust bi‐directional replays during phase 2.

#### 2.1.2 Phase 2 Offline Replay

The PC‐to‐PC connections, and weights learned during Phase 1, were carried to Phase 2 for the offline simulations. Following Ecker et al. (2022), the network architecture was further expanded during the offline phase to include 150 inhibitory BCs and 8000 stochastic mossy fiber (MF) cells. All PC and BC neurons in these populations were modeled as adaptive integrate‐and‐fire (Ad‐IF) units, with spiking driven by synaptic input rather than the imposed spiking patterns delivered during Phase 1. The MF cells provide excitatory input to the PC population during Phase 2 and comprised the sole external input to the network during this phase. These MF cells were modeled as independent Poisson neurons spiking at rate $\lambda_{\mathrm{MF}}=0.1$. The MF population was connected 1:1 to the PC population with strong “detonator” synaptic weights (19.15 nS). Connections between the BC neurons and the other nodes in the network were probabilistic, with connection probabilities set as follows: 0.1 for BC‐to‐PC, 0.25 for PC‐to‐BC, and 0.25 for BC‐to‐BC connections. BC and MF cells were not connected.

As in the Ecker et al. (2022) model, during Phase 2 the PC and BC populations were modeled as:

(4) $$C_{m}\frac{\mathrm{dV}\left(t\right)}{\mathrm{dt}}=-g_{L}\left(V\left(t\right)-V_{\text{rest}}-\Delta_{T}\cdot e^{\frac{\left(V\left(t\right)-\theta \right)}{\Delta_{T}}}\right)+I_{\mathrm{syn}}\left(t\right)+\mathrm{ad}\left(t\right)$$

(5) $$\frac{d\;\mathrm{ad}\left(t\right)}{\mathrm{dt}}=\frac{c\left(V\left(t\right)-V_{\text{rest}}\right)-\mathrm{ad}\left(t\right)}{\tau_{\mathrm{ad}}}+b\sum_{k}\delta \;\left(t-t_{k}\right)$$

where: $C_{m}$ is the membrane capacitance; $g_{L}$ is the leak conductance; $V_{\text{rest}}$ is the reversal potential; θ is the intrinsic spike threshold; $\Delta_{T}$ is the slope factor (See Brette and Gerstner 2005); $I_{\mathrm{syn}}$ is the synaptic current (See Equation 6); here $\mathrm{ad}\left(t\right)$ is an adaptation current, $t_{k}$ denotes the k‐th spike time, and the Dirac delta term $b\sum_{k}\delta \left(t-t_{k}\right)$ produces an instantaneous increment ad→ad+b at each spike.

For pyramidal cells (PCs), parameters were

$$b=206.9\;\mathrm{pA},\;c=-0.27\;\mathrm{nS},\;\tau_{\mathrm{ad}}=85\;\mathrm{ms}.$$

For BCs, parameters were

$$b=0.91\;\mathrm{pA},\;c=3.05\;\mathrm{nS},\;\tau_{\mathrm{ad}}=178.6\;\mathrm{ms}.$$

The negative value of c in PCs reflects dominance of the spike‐triggered adaptation component, whereas in BCs the positive c introduces stronger voltage‐dependent adaptation. These parameter regimes were adopted from Ecker et al. (2022), in which they were calibrated to reproduce physiologically realistic spike‐frequency adaptation and stable replay dynamics in CA3 network simulations.

The total synaptic current $I_{\mathrm{syn}}\left(t\right)$ received by a neuron is modeled as the sum of excitatory and inhibitory components:

(6) $$I_{\mathrm{syn}}\left(t\right)=g_{\mathrm{PC}}\left(t\right)\left(V\left(t\right)-E_{\mathrm{exc}}\right)+g_{\mathrm{BC}}\left(t\right)\left(V\left(t\right)-E_{\mathrm{inh}}\right)$$

where $E_{\mathrm{exc}}=0\;\mathrm{mV}$ and $E_{\mathrm{inh}}=-70\;\mathrm{mV}$ are the reversal potentials for excitatory and inhibitory synapses, respectively. The total conductance from each input population (e.g., PCs for excitation, BCs for inhibition) is computed by summing the postsynaptic response to each presynaptic spike:

(7) $$\begin{array}{c} g_{X}\left(t\right)=\sum_{j\in P_{X}}\sum_{k}\;{\hat{g}}_{j}\cdot Q\cdot \left[\exp\left(-\frac{t-t_{j}^{\left(k\right)}-d_{j}}{\tau_{d}}\right)-\exp\left(-\frac{t-t_{j}^{\left(k\right)}-d_{j}}{\tau_{r}}\right)\right]\\ \;\cdot \;1\left\{t-t_{j}^{\left(k\right)}-d_{j}\geq 0\right\} \end{array}$$

here $X\in \left\{\mathrm{PC},\mathrm{BC}\right\}$ denotes the presynaptic population (excitatory or inhibitory), $P_{X}$ is the set of presynaptic neurons of type X, $t_{j}^{\left(k\right)}$ is the time of the $k^{\mathrm{th}}$ spike from neuron j, $d_{j}$ is the synaptic delay from neuron j, ${\hat{g}}_{j}$ is the peak conductance of the synapse from neuron j, $\tau_{r}$, and $\tau_{d}$ are the rise and decay time constants, $-Q={\left(\exp\left(-\frac{t_{\text{peak}}}{\tau_{d}}\right)-\exp\left(-\frac{t_{\text{peak}}}{\tau_{r}}\right)\right)}^{-1}$ normalizes the peak conductance to ${\hat{g}}_{j}$, with $t_{\text{peak}}$ given by:

(8) $$t_{\text{peak}}=\frac{\tau_{r}\tau_{d}}{\tau_{d}-\tau_{r}}\ln\left(\frac{\tau_{d}}{\tau_{r}}\right).$$

The indicator function component $1\left\{t-t_{j}^{\left(k\right)}-d_{j}\geq 0\right\}$ ensures causality in the synaptic response: the conductance change becomes nonzero only after the corresponding presynaptic spike has occurred and its associated synaptic delay has elapsed.

#### 2.1.3 Plasticity During Phase 2

In the Ecker et al. (2022) model, spontaneous bi‐directional replays are observed during the offline state, interspersed with periods of sparse and unstructured activity (nonreplay periods). In contrast with the absence of offline plasticity in Ecker et al. (2022), we incorporated different synaptic plasticity rules specifically in the PC‐to‐PC recurrent synapses. To enable selective activation of different plasticity kernels, we introduced the variable p into the Phase 1 plasticity rule of Ecker et al. (2022). Consequently, our synaptic weight adaptation rule is defined as:

(9) $$\Delta w_{+}=p_{+}\cdot A_{+}\exp\left(\frac{-\Delta t}{\tau^{+}}\right)\;\mathrm{at}\;t_{\text{post}}\;\text{if}\;\left(t_{\mathrm{pre}}+d\right)<t_{\text{post}}$$

(10) $$\Delta w_{-}=p_{-}\cdot A_{-}\exp\left(\frac{\Delta t}{\tau^{-}}\right)\;\mathrm{at}\;t_{\mathrm{pre}}\;\text{if}\;\left(t_{\mathrm{pre}}+d\right)>t_{\text{post}}$$

where.

$p_{+/-}$ is the multiplier used to activate PKCs, ranging from −1 to 1, $\tau_{+/-}$ is the decay time constant, d is the synaptic delay (set to 2.2 ms for PC‐to‐PC synapses), $t_{\mathrm{pre}}$ and $t_{\text{post}}$ are the times of presynaptic and postsynaptic action potentials, respectively, $\Delta t=t_{\text{post}}-\left(t_{\mathrm{pre}}+d\right)$ represents the time difference between the postsynaptic and presynaptic action potentials (accounting for synaptic delay), and $A_{+/-}$ is the amplitude of the weight update in nS, which decays exponentially with the respective time constant $\tau_{+/-}$.

Note: Except for the introduction of plasticity during Phase 2, we retained the parameter configuration of the Ecker et al. (2022) offline state model, including synaptic modeling, synaptic delays, special treatment of track edges, and all other implementation details. For further information, see Ecker et al. (2022).

### 2.2 Simulation Parameters

#### 2.2.1 PKC Parameters

In each simulation, we activated one PKC from the presynaptic‐fires‐first group (PT‐P or PT‐D) and one PKC from the postsynaptic‐fires‐first group (NT‐P or NT‐D). The only exception was during our replay speed investigation, where we activated a single PKC in some simulations to assess its contribution to replay speed modulation.

The time decay constant (τ) was uniformly sampled from the range [5, 30 ms], and the change amplitude of synaptic connection strength ($A_{+/-}$) was uniformly sampled from the range [0.005, 0.05 nS]. These parameter ranges were selected based on the initial training parameters used in the Ecker et al. (2022) model for the asymmetric STDP rule (τ=20, $A_{+/-}=0.04$ nS).

Unless otherwise specified, PKCs had identical time and amplitude size components (e.g., $\tau_{+}=\tau_{-}$ and $|A_{+}|\;=\;|A_{-}|$). Consequently, during hSTDP or anti‐hSTDP simulations, the synaptic strengthening and weakening PKCs were of equal size.

#### 2.2.2 Simulation Setup

Unless otherwise noted, we varied the initial conditions of our simulations by re‐running Phase 1 to generate a new PC‐to‐PC synaptic weight matrix before each offline phase simulation. This ensured that each offline phase simulation started with a different (though statistically similar) synaptic configuration.

We conducted 100 simulations using asymmetric learning rules ($A_{+}=-A_{-}$) and 50 simulations with a symmetric kernel ($A_{+}=A_{-}$), covering a range of plasticity kernel sizes. The asymmetric simulations were run for 15 s to allow observation of replay pattern progression, especially under smaller plasticity kernels. The symmetric kernel simulations were run for 10 s, matching the simulation duration in Ecker et al. (2022) and providing sufficient time to categorize replay progression.

### 2.3 Simulation Data Collection

During each simulation, we recorded spike times of PCs and BCs using Brian2's (Stimberg et al. 2019) spike and state monitoring tools. The simulation outputs—including neuron spike times, simulation parameters, and synaptic weight changes—were stored in a Microsoft SQL Server^1^ database for analysis. We calculated the spiking rate for each neuron based on its interspike interval (ISI), defined as the time between the current and previous spike of the same neuron. For example, if a PC spikes twice within 100 ms, a firing rate of 10 Hz is assigned to the second spike. Each simulation was also associated with key parameters: $\tau_{+}$ and $\tau_{-}$ (taup and taum); $A_{+}$ and $A_{-}$ (Ap and Am); and a unique experimental ID.

#### 2.3.1 Data Analysis Tool

**FIGURE 2:**
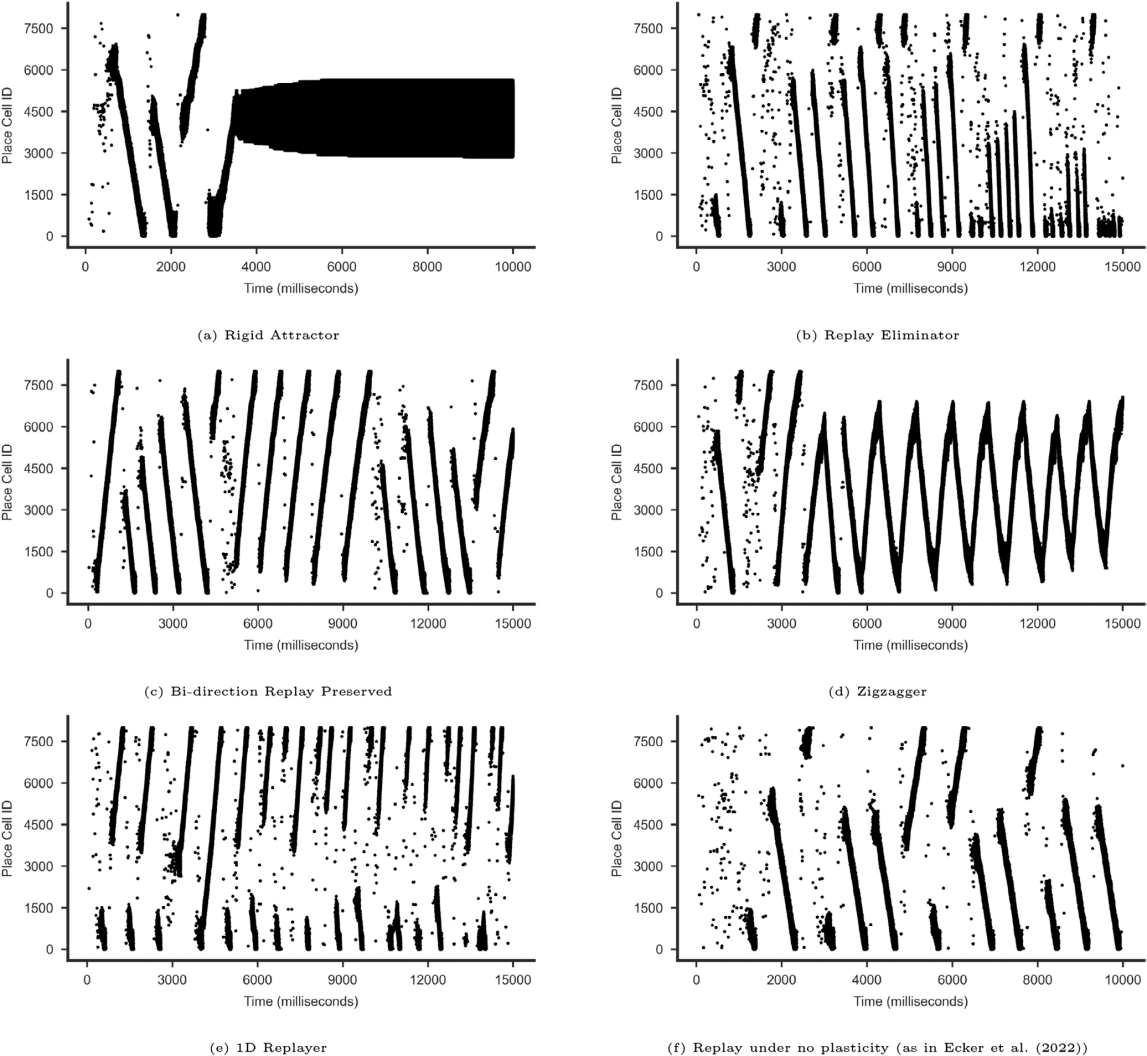
Example patterns observed in simulations of replay progressions. (a–f) Raster plots from sample simulations displaying typical replay progression behaviors. (a)–(d) are 20 s long. (e and f) are 15 s long. A minimum 10 Hz filter was applied to the raster plots to remove noisy nonreplay spiking periods. X axis: Timeline in milliseconds (ms) from simulation start, Y axis: PC IDs. f) No plasticity is the replay pattern in the Ecker et al. (2022) paper. See additional examples at Figures A1, A2, A3.

We developed an interactive data analysis dashboard using Power BI^2^ to support spiking pattern analyses and replay identification. Spiking activity of the PC population was visualized via raster plots, with time (ms) on the X‐axis and PC IDs (0‐7999) on the Y‐axis. We applied ISI‐based spike rate filters, as in Ecker et al. (2022), to highlight periods of elevated PC activity and suppress background noise, enabling clear visual identification of replay events (Figure 2). This visual analysis approach played a key role in guiding and validating the parameter choices used in our statistical analyses.

### 2.4 Replay Detection

We visually identified replay events by fitting a regression line to the spiking activity, using time (in milliseconds) as the X‐axis and PC ID as the Y‐axis. An event was classified as a replay if the absolute value of the regression slope was at least 1.5, ensuring that the activation sequence exhibited the characteristic smooth progression observed in our visual analyses. Additionally, to be considered a replay, the event had to span at least 1500 PCs ($\frac{3}{16}$ of the track) and last at least 150 milliseconds, as events with shorter distance and/or duration often lacked the structured sequential activation pattern necessary to meet our replay definition.

### 2.5 Replay Candidate Identification and Validation

Unlike the Ecker et al. (2022) model, our offline simulation phase altered PC firing patterns, requiring a revised approach to replay detection. Rather than using their criterion—PC population firing above 2 Hz for at least 260 ms—we identified replay candidates based on BC activity, which was appropriate given that PC‐to‐BC and BC‐to‐BC synaptic strengths were fixed at 0.65 and 5 nS, respectively. Using ISI‐based spike rate calculations, we defined a replay candidate as any period in which at least five BCs fired at 120 Hz or more within a 10 ms window. The candidate period ended once this threshold was no longer met. To filter out short, noisy events, we required a minimum candidate duration of 150 ms. We validated replay candidates by assessing sequential PC activation. Specifically, we located the median PC active during the candidate period and extracted a 500‐PC segment centered around it (±250 PCs). A linear regression was then applied over this segment, and candidates were confirmed as replays only if the resulting slope met our predefined criteria. All validated replay events were stored in our results database, along with experiment ID, replay start and end timestamps (in milliseconds), 500‐PC segment bounds, segment activity duration, and the calculated regression slope.

### 2.6 Replay Speed Calculation

To quantify replay speed, we focused on the central portion of each replay event. We identified the median PC active during the replay and selected a 500‐PC segment centered around it (±250 PCs). This approach helped avoid the variability typically observed during replay initiation and termination (i.e., the initial and terminal 500‐PC segments which showed inconsistent speeds during inspection).

We noted that typical replays emerged from an active subpopulation of approximately 200 PCs at a random location and required a variable ramp‐up period before propagating toward the track end. At the track boundary, activity often lingered for inconsistent durations before dissipating. For these reasons, we excluded candidate events that spanned less than 3/16 of the track from our speed analysis, as they frequently exhibited irregular propagation speeds. We used two methods to assess replay speed:

#### 2.6.1 Duration‐Based Speed Measurement

We defined replay duration as the interval between the first and last milliseconds during which at least three PCs were co‐active (in 1‐ms bins) within the 500‐PC segment. This method measured the full duration of elevated activity in the segment. The three‐PCs threshold allowed us to filter out nonreplay activity generated by the stochastic MF input.

#### 2.6.2 Regression Slope Speed Measurement

As an alternative metric, we computed the slope of a regression line fitted to PC spiking in the 500‐PC segment, with time (ms) on the X‐axis and PC ID on the Y‐axis. This provided a measure of replay direction (positive for forward, negative for reverse) and captured the time it took for activity to propagate across the segment. However, this method did not account for the tail‐end dissipation of activity and sometimes yielded higher replay speeds than the duration‐based method.

#### 2.6.3 Plasticity Kernel Contribution to Replay Speed Analysis

To investigate the impact of plasticity on replay speed, we fixed the kernel parameters and starting PC‐PC weight matrix ($\tau_{+/-}$ = 10 ms and $|A_{+/-}|$ = 0.15 nS) to isolate the influence of the PKC activation scheme. We conducted 200 simulations with single PKC activation (50 simulations for each PKC) and 200 simulations with dual PKC activation (50 simulations for each combination: hSTDP, anti‐hSTDP, st‐LTP, and st‐LTD). We used the duration‐based method to assess changes in replay speed over time, fitted with quadratic curves. The simulations for replay speed analysis were 10 s long, which was the original simulation duration used by Ecker et al. (2022).

### 2.7 Replay Progression Pattern Classification

Visual analysis of simulation results identified several distinctive patterns. We therefore developed a categorization framework for these simulation outcomes based on the frequency, directionality, and length of replays over time. Some simulations exhibited elevated firing rates in both PCs and BCs as the simulation progressed, without transitioning back to a nonreplay state. We classified such simulations as resulting in *saturated activity*, which was further divided into two subtypes:

- *Rigid Attractor:* Characterized by rigid, high‐level activity that persists without variability (Figure 2a).
- *Zigzagger:* Characterized by a dynamic center of activity that switches directions frequently and often does not terminate at the track ends (Figure 2d).

In other simulations, the network continued to generate replays. These simulations followed two types of patterns which we categorized as:

- *Directionally Biased:* Characterized by reverse replays in the track start section (lower part of raster plot) and forward replays in the track end section (upper part of raster plot) (Figure 2e).
- *Bidirectional:* Characterized by longer/more complete replays in both directions with periodical switching between forward and reverse replays (Figure 2c).

All classification parameters described in the following were validated using our visual analysis tool.

#### 2.7.1 Saturated Activity: Rigid Attractor and Zigzagger

In each simulation, we divided the last 3000 into 25 ms time bins (120 total segments) and measured two metrics within each segment: the number of BC spikes and the slope of the PC spiking pattern. A segment was flagged as a candidate for saturated activity if it contained more than 50 BC spikes. If the number of saturated segments exceeded 100 (i.e., > 83.3% of the last 3000 ms), we then categorized the simulation end state as either *Rigid Attractor* or *Zigzagger* based on the following criteria:

- If the average number of BC spikes per segment was >700, the simulation was classified as *Rigid Attractor*.
- If the average BC spikes per segment was <700, we searched for the existence of short replays (approximately 100 ms long) in both directions. Specifically, we looked for at least four consecutive segments where the slope was consistently in the forward direction (slope >1) or the reverse direction (slope <−1).
- If such short replays were found in both directions, the simulation was classified as *Zigzagger*.
- If neither condition was met, the simulation was categorized using one of the nonsaturated end‐state definitions described below.

#### 2.7.2 Replay Preserved: Directionally Biased and Bidirectional

We classified a simulation as “replay preserved” if it exhibited at least one replay spanning more than 1500 PCs and lasting over 100 ms during the final 2000 ms of the simulation. Additionally, we required at least 200 ms of nonreplay activity during the last 3000 ms of the simulation to exclude saturated activity. The simulation end state classification was applied only after the first 5000 ms of the simulation, allowing time for plasticity effects to stabilize.

##### 2.7.2.1 Directionally Biased

Simulations were categorized as directionally biased if more than 80% of replays in a given track segment occurred in the same direction (forward or reverse). Since in general the top portion of the track (PC IDs > 4000) showed a tendency toward forward replay while the bottom portion (PC IDs < 4000) showed a tendency toward reverse replay, only replays that (i) covered at least 50% of the track and (ii) began after 5000 ms were considered. Shorter replays that did not cross the track midpoint were excluded due to these apparent directional biases.

##### 2.7.2.2 Bi‐Directional Replay Preserved (BiDRP)

Simulations were classified as BiDRP when the replays alternated spontaneously between directions—typically 2–5 consecutive replays in one direction followed by a similar number in the opposite direction. To qualify as BiDRP, simulations had to exhibit replays in both directions, each spanning at least 50% of the track and starting after 5000 ms.

#### 2.7.3 Replay Eliminator

A simulation in which the network ceased generating replays during the last 2000 ms was categorized as “Replay Eliminator” (Figure 2b).

### 2.8 hSTDP and Anti‐hSTDP With Unequal PKCs Size

We ran a total of 100 simulations with uneven Kernels while preserving an asymmetric rule (e.g., $\mathrm{sign}\left(A_{+}\right)\neq \mathrm{sign}\left(A_{-}\right)$, $A_{+}\neq {A_{-}}^{*}\left(-1\right)$, and $\tau_{+}\neq \tau_{-}$). 51 simulations with co‐activated hSTDP and 47 simulations with co‐activated anti‐hSTDP. We used an identical initial PCs weight matrix for all simulations and ran the simulation for 15 s. We randomly selected the values for $A_{+},A_{-}$ from ±0.005 to ±0.05 nS range and $\tau_{+},\tau_{-}$ from 5 to 30 ms range. To plot our results (Figure 7), we calculated each PKC size using the following equations: $\tau_{+/-}$ is the decay time constant in ms; $A_{+/-}$ is the amplitude of the synaptic weight change in nS, which decays exponentially, Δt is time difference in ms between the postsynaptic spike and the presynaptic spike. The units of PKC size are nS*ms.

## 3 Results

### 3.1 Bidirectional Replays Maintained With Plasticity Off

**FIGURE 3:**
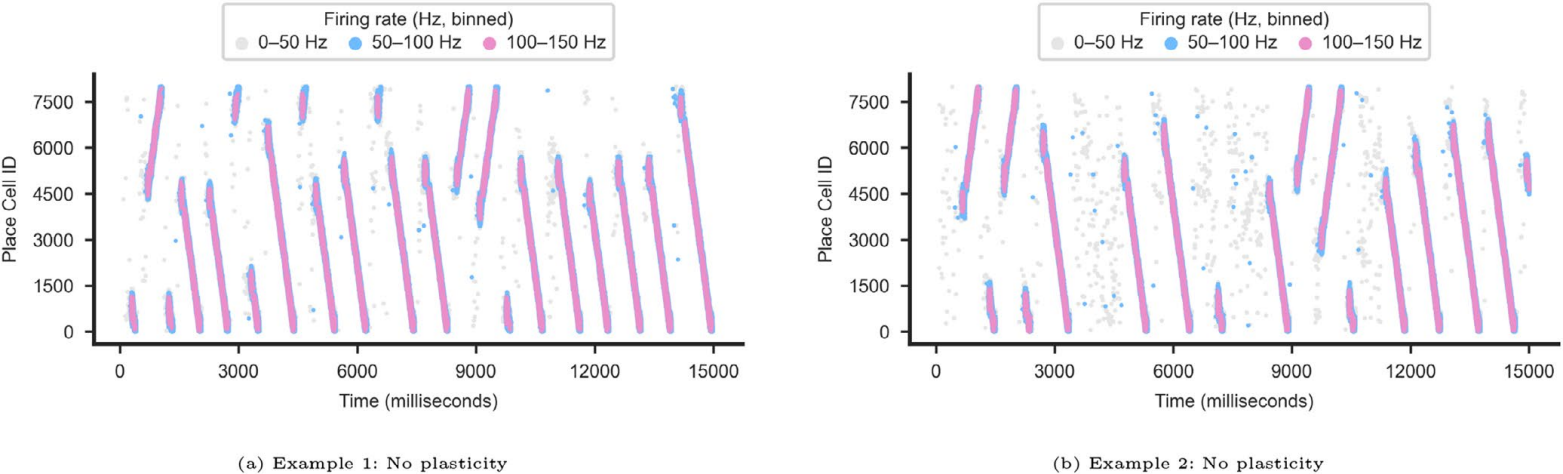
Examples of typical replay patterns generated when plasticity was turned off during the offline phase. (a and b) Raster plots from sample 15‐s simulations displaying typical replay progression behaviors. X axis: Timeline in milliseconds (ms) from simulation start, Y axis: Place cell IDs. Legend (Spiking rate): Gray 0–50 Hz, light blue 50–100 Hz, Pink 100–150 Hz.

We were able to run the original code from Ecker et al. (2022) without plasticity and confirm that we could produce replay pattern similar to their original results (Figures 2f, 3a,b). The replays were originating at random points along the track and progressing either in the forward or reverse direction until terminating at the respective track end. Thus, we were able to replicate Ecker et al. (2022)'s results with our modified model when we set kernel parameters $A_{+}$ and $A_{-}$ to zero.

### 3.2 Impact of Symmetric Plasticity (St‐LTP and St‐LTD) on Replay Dynamics

In Ecker et al. (2022), a symmetric Hebbian plasticity rule was used during learning to generate a robust mix of forward and reverse replays. However, this plasticity rule was turned off during off‐line simulations, which is not physiologically realistic, though the exact plasticity rules in effect during off‐line periods remain unknown. Indeed, both synaptic potentiation and synaptic depression have been associated with sleep (Rasch and Born 2013; Tononi and Cirelli 2014). To better understand how plasticity impacts the dynamics of replays in the network, we performed simulations under different potential plasticity rules.

**FIGURE 4:**
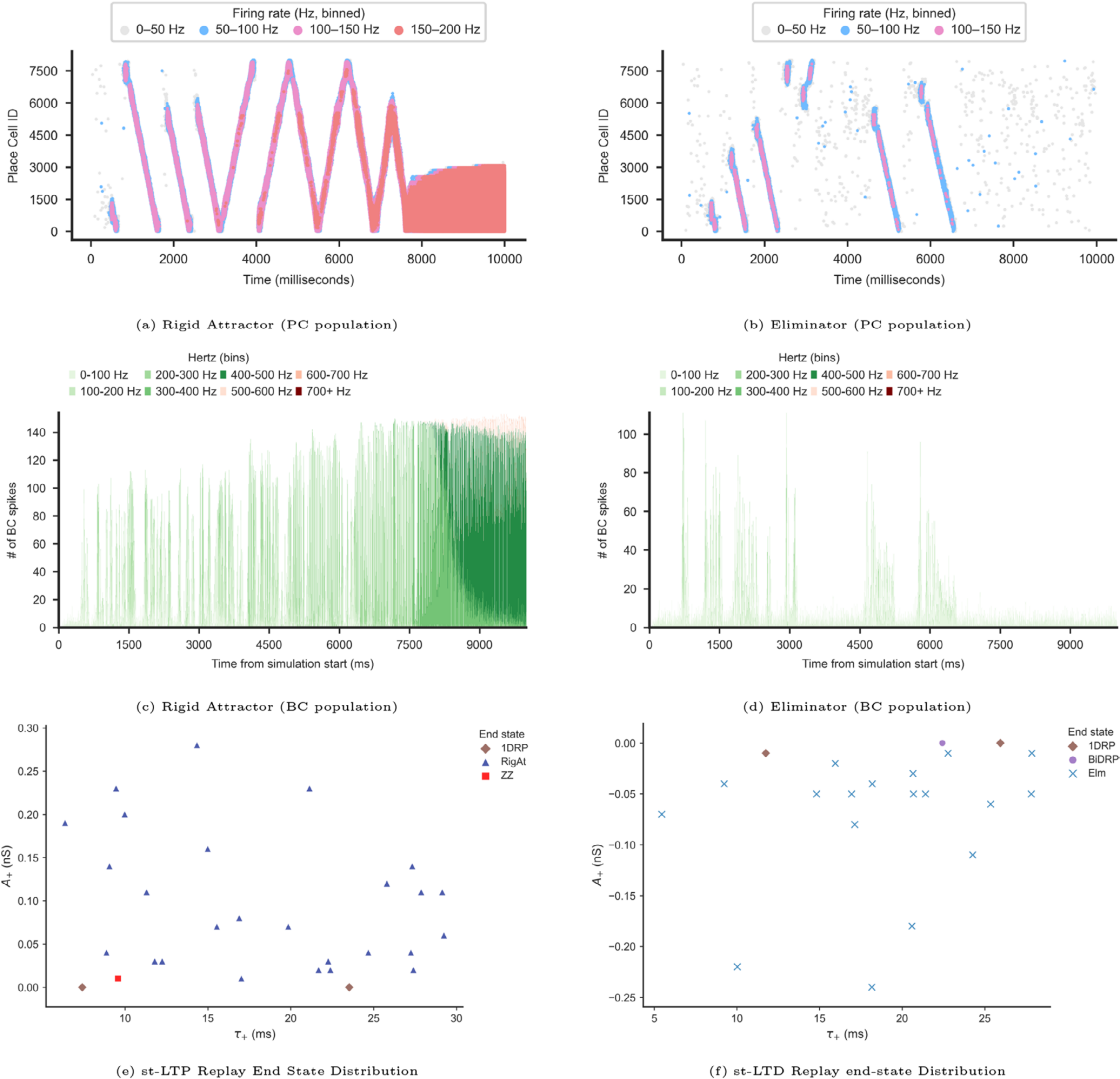
Impact of st‐LTP and st‐LTD on replay dynamics. (a and b) Raster plots from representative simulations showing typical replay progression. X‐axis: Time (ms) from simulation start. Y‐axis: Place Cell ID. Each mark indicates a spike; color represents firing rate (Hz)—gray: 10–50 Hz, light blue: 50–100 Hz, pink: 100–150 Hz, red: > 150 Hz (see Section 2). (c and d) BC population spike histograms (2 ms bins). X‐axis: Time (ms). Y‐axis: BC spike count in the 10 ms bin. Legend: Firing rate (Hz) bins (100 Hz size). (e and f) Simulation end state classification (see Section 2). Each mark represent a 10 s simulation end state. X‐axis: $\tau_{+}$; Y‐axis: $A_{+}$. Legend—blue triangle: Rigid Attractor, red square: Zigzagger, brown diamond: 1D Replayer, light blue X: Eliminator, purple circle: Bi‐directional Replay Preserved All simulations ran for 10 s.

First, we examined the scenario in which both $A_{+}$ and $A_{-}$ were positive, corresponding to PT‐P and NT‐P, which we refer to as st‐LTP (time‐dependent long‐term potentiation. Figure 1b—Upper right panel). As expected with such a potentiation‐biased rule, in these simulations, synaptic weights increased with each successive replay (Figure 4a and Figure A3a,b). We still observed bi‐directional replays in the early stages of these simulations, similar to under the no‐plasticity regime Ecker et al. (2022). However, continued replays naturally led to further potentiation of synaptic connections, which resulted in increasingly frequent propagating replays in alternating directions (e.g., a replay in the forward direction that was immediately followed by a replay in the reverse direction). The progressive strengthening resulted in what we termed a “Rigid Attractor” state, characterized by a subpopulation of the principal neurons firing at high instantaneous frequencies (over 100 Hz). This overactive subpopulation strongly activated the BC population while suppressing activity in the rest of the PC network via feedback inhibition (Figure 4c). Our simulation often resulted in a “Rigid Attractor” state even when small kernel values were used (Figure 4e).

We next examined activation of the plasticity kernel with $A_{+},A_{-}<0$, mimicking net synaptic depression, with PT‐D and NT‐D which in combination we refer to as st‐LTD (time‐dependent long‐term depression (LTD). Figure 1b—Lower right panel), which can take place at different synapses under specific neuromodulatory conditions (Debanne and Inglebert 2023) and has been hypothesized for the sleep state (Tononi and Cirelli 2014; Vyazovskiy et al. 2008). In these simulations, replays disappeared after only a few occurrences, even under relatively small plasticity kernels (Figures 4b,d,f and A3c in the Appendix A). Thus, not surprisingly, strong synaptic depression led to erasure of the original learned trace in the network. These results highlight the importance of the network's synaptic weight structure on replay generation.

### 3.3 Hebbian STDP Induces Directional Bias and Progressive Replay Degradation

**FIGURE 5:**
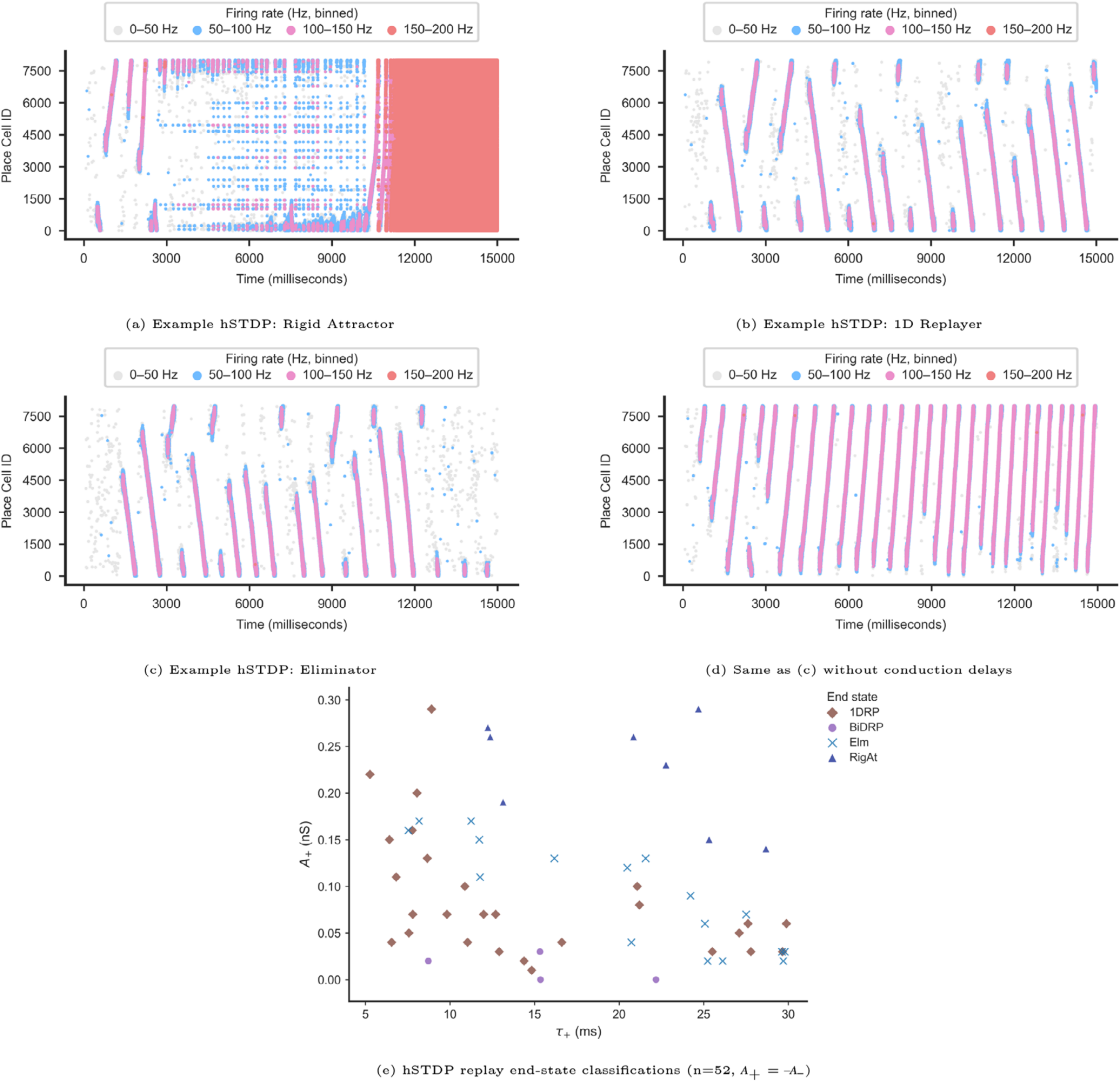
Examples of replay patterns generated when hSTDP plasticity was applied during the offline phase simulation. (a–d) Raster plots from sample simulations displaying typical replay progression behaviors. All simulations are 15 s long. (c) and (d) are identical $A_{+}$ and $\tau_{+}$ with synaptic delay is set to 0 at d and 2.2 ms at (c). X axis: Timeline in milliseconds (ms) from simulation start, Y axis: Place cell IDs. Color Legend (Spiking rate): Gray 0–50 Hz, light blue 50–100 Hz, Pink 100–150 Hz, Red 150+ Hz. (e) Impact of PKC Parameters $A_{+/-}$ and $\tau_{+/-}$ (see Equations 9 and 10) on replay progression patterns. Only $A_{+}$ and $\tau_{+}$ are shown in (e), where $\tau_{+}=\tau_{-}$ and $A_{+}=-A_{-}$. Before each simulation, PC‐to‐PC connections were re‐initialized by re‐running the phase 1 simulation, which includes probabilistic starting conditions. Axes: X‐axis represents $\tau_{+}$ in milliseconds (ms); Y‐axis represents $A_{+}$ in nano‐Siemens (nS). Legend: Dark blue triangle represents Rigid Attractor (RigAt), light blue “x” represents Eliminator (Elm.), purple circle represents Bi‐directional Replay Preserved (BiDRP), and brown diamond represents 1D Replayer.

**FIGURE A1:**
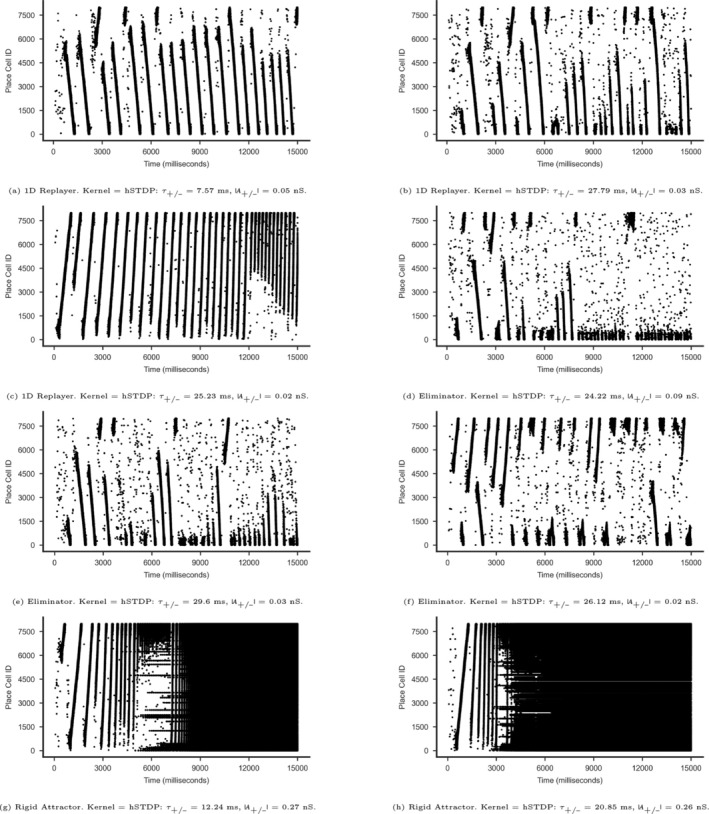
Additional examples of our simulation categorization methodology. (a–h) Raster plots of PCs' spiking pattern progression under different plasticity kernels. Each dot represents a PC spike. Minimum spiking rate of 10 Hz. Y Axis—PC ID number, X Axis—Time from simulation start (in ms).

Next, we performed simulations under more balanced STDP rules with both a strengthening and a weakening kernel component. We found that distinct patterns emerged under the protoypical hSTDP kernel (i.e., with PT‐P and NT‐D. Figure 1b—Upper left panel); reverse replays dominated the lower part of the raster plot (track start) and forward replays dominated the upper part (track end), but very few replays spanned the entire track (Figures 5b,c and A1a–c). These patterns indicated a “demarcation zone” around the middle of the track above which replays were predominantly forward and below which they were predominantly reverse. The location of the “demarcation zone” was typically decided by the starting points of the first few replays in the simulation. Under the hSTDP plasticity kernel, replay activities were largely confined to the far track ends (Figures 5c and A1d–f), with very few track segments participating in any bi‐directional replays.

**FIGURE A2:**
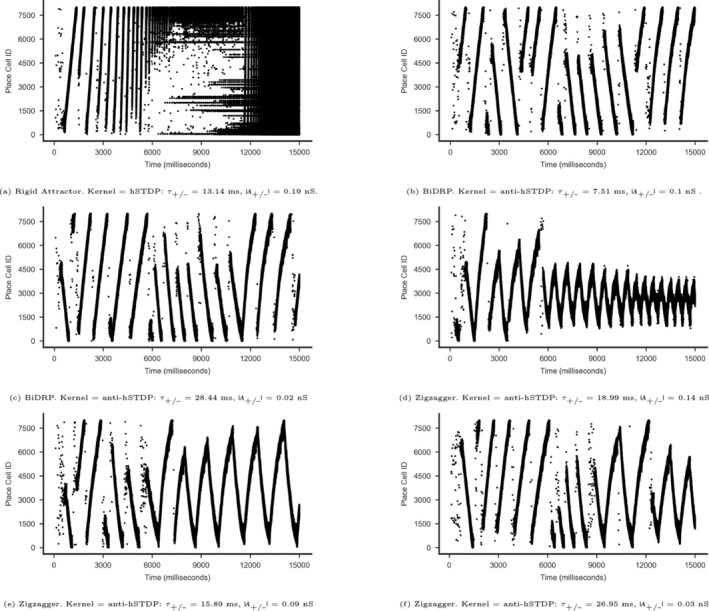
Additional examples of our simulation categorization methodology. (a–f) Raster plots of PCs' spiking pattern progression under different plasticity kernels. Each dot represents a PC spike. Minimum spiking rate of 10 Hz. Y Axis—PC ID number, X Axis—Time from simulation start (in ms).

**FIGURE A3:**
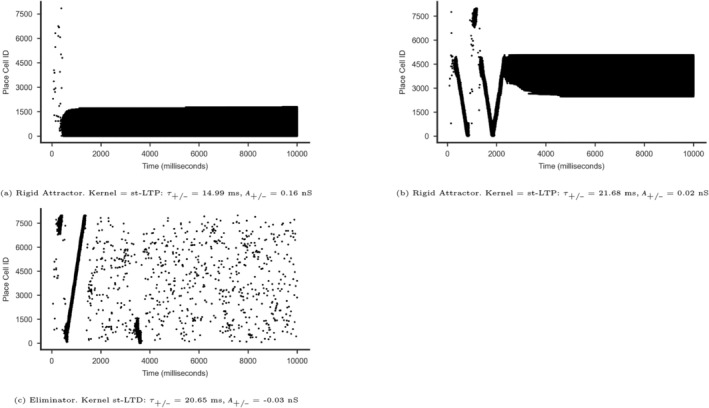
Additional examples of our simulation categorization methodology. (a–c) Raster plots of PCs' spiking pattern progression under different plasticity kernels. Each dot represents a PC spike. Minimum spiking rate of 10 Hz. Y Axis—PC ID number, X Axis—Time from simulation start (in ms).

To better understand the relationship between hSTDP kernel selection and the resulting replay behaviors, we performed a parameter sweep of different kernel sizes and assessed the results using our classification criteria 2.7. Our analysis showed (Figure 5e) that with smaller kernels ($|A_{+/-}|\;<0.15$ nS and $\tau_{+/-}<20$ ms), replay activity progressively degraded, with the network generating fewer and/or shorter replays over time. In many simulations, replays ceased entirely within the final 2 s used for classification (see Figure 5c and Section 2). However, some simulations still produced one or two late replays, suggesting that activity may not be fully eliminated within the 15‐s simulation window. Extending simulation durations led to reclassification of several *1D Replayer* cases as *Eliminator* cases. In contrast, larger kernels ($|A_{+/-}|\;>0.15$ nS and $\tau_{+/-}>12$ ms) resulted in saturated firing consistent with Rigid Attractor states (Figure 5a). We also noted that unlike the Rigid Attractors produced by st‐LTP (Figure 4a) that were contained in a continuous subsection of the track, Rigid Attractors produced by large hSTDP kernels were not continuous and appeared as high‐frequency noise (Figures 5a and A1g,h, A2a).

Based on these observations, we concluded that following an initial replay event, arising spontaneously at a random starting location, subsequent replays were prone to follow the same direction due to strengthening of the corresponding synaptic weights. Over time, this directional bias continued to strengthen. Successive replays in the same direction further led to a progressive acceleration of the replays in this direction (Figure 5c,d). This finding prompted a more detailed investigation into the impact of plasticity on replay speed (which we focus on in Section 3.6).

Given that simulations with smaller kernels frequently resulted in the Eliminator end‐state, we hypothesized that smaller kernels may yield “decoupling by synchrony” (Lubenov and Siapas 2008), wherein connections between highly synchronized neurons undergo LTD resulting from a combination of STDP and conduction delays (see Lubenov and Siapas (2008) for details). To test whether synaptic delays resulted in Eliminator states, in accordance with this hypothesis, we re‐ran simulations with smaller hSTDP kernels while setting the PC‐to‐PC synaptic delays to zero (see Figure 5c,d for comparison). As we expected, without synaptic delays, replays were not eliminated but instead accelerated until becoming highly synchronized. These observations thus support the critical role of synaptic delays in replay degradation under hSTDP.

### 3.4 Offline Activation of Anti‐hSTDP Preserves Bi‐Directional Replay

**FIGURE 6:**
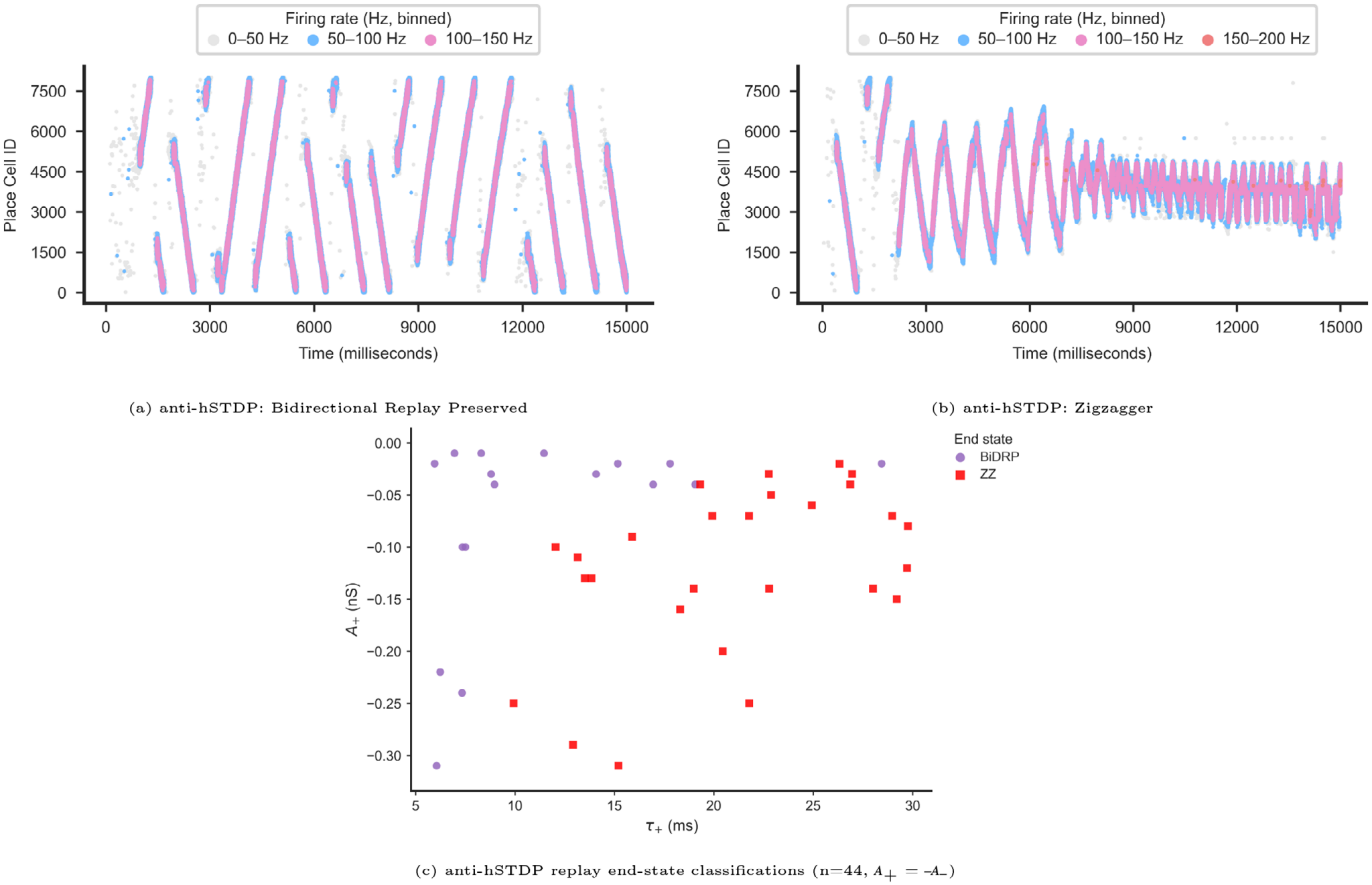
Examples of replay patterns generated when hSTDP plasticity was applied during the offline phase simulation. (a and b) Raster plots from sample simulations displaying typical replay progression behaviors. All simulations are 15 s long. X axis: Timeline in milliseconds (ms) from simulation start, Y axis: Place cell IDs. Color Legend (Spiking rate): Gray 0–50 Hz, light blue 50–100 Hz, Pink 100–150 Hz, dark blue 150+ Hz. (c) Impact of PKC Parameters $A_{+/-}$ and $\tau_{+/-}$ (see Equations 9 and 10) on replay progression patterns. Only $A_{+}$ and $\tau_{+}$ are shown in (c), where $\tau_{+}=\tau_{-}$ and $A_{+}=-A_{-}$. Before each simulation, PC‐to‐PC connections were re‐initialized by re‐running the phase 1 simulation, which includes probabilistic starting conditions. Axes: X‐axis represents $\tau_{+}$ in milliseconds (ms); Y‐axis represents $A_{+}$ in nano‐Siemens (nS). Legend: Red square represents Zigzagger (ZZ), Purple circle represents Bidirectional Replayer (BiDRP).

In addition to hSTDP, we sought to understand the impact that an anti‐hSTDP kernel, with PT‐D and NT‐P as described by Pandey and Sikdar (2014) (Figure 1b—Lower left panel), would have on replay structure and progression. Similar to hSTDP, this kernel is also balanced, with equally sized positive (LTP) and negative (LTD) components. In these simulations, we observed activation patterns very different from those under hSTDP. While the first few replay events resembled the postlearning ones as in Ecker et al. (2022), later replays appeared more complete, covering a greater portion of the track in each given event, with periodical shifts between the forward and reverse directions (Figures 6a and A2b,c). We further noticed that as the simulations progressed, the durations of interreplay periods diminished over time. We suspected that the selection of kernel size parameters was the main driver behind the increased frequency of replay. Crucially, we also observed stabilization in the propagation speed of replays. Replay speeds increased or decreased with each periodic change in replay direction, decelerating during consecutive replays in one direction and accelerating when the replay direction switched (see Section 2). This suggests that reversals of replay direction allowed for replay speed to remain constrained within specific limits. We explore this point further in Section 3.6 below. When we selected a larger anti‐hSTDP kernel, we observed that the model began to generate continuous replays in alternating directions (Figures 6a and A2b).

This finding motivated our parameter sweep analysis to investigate the relationship between kernel parameter selection and the resulting replay structure (Figure 6c). Our analysis revealed that for small Kernels ($|A_{+/-}|\;<0.15$ nS, $\tau_{+/-}<15$ ms), bi‐directional replay activities were preserved (Figure 6a) while for larger Kernels ($|A_{+/-}|\;>0.15$ nS, $\tau_{+/-}>15$ ms), the network consistently generated replays without nonreplay periods. Replay events and direction changes became more frequent, eventually forming a Zigzag pattern (Figure 6b), where a replay in one direction was immediately followed by a replay in the opposite direction. Zigzagger simulations often displayed progressively shorter replays, which often did not arrive at the ends of the track.

Based on these analyses, we conclude that the replay dynamics under the anti‐hSTDP kernel are driven by the interaction between synaptic weakening in the direction of the replay and simultaneous strengthening of retrograde connections. As a replay propagates in one direction, it decreases the likelihood of future replays following the same path while increasing the probability of replays in the opposite direction, causing a periodical shift in prevailing replay direction. In addition, the interplay between spontaneous replay initiation sites and the evolving synaptic potentiation gradually shifts the origin of replays toward the ends of the track (as seen in Figure A2b,c). Thus, replays under anti‐hSTDP kernels are uniquely able to preserve the original memory trace.

### 3.5 Uneven PKCs in Asymmetric STDP Rapidly Degrade Replays

**FIGURE 7:**
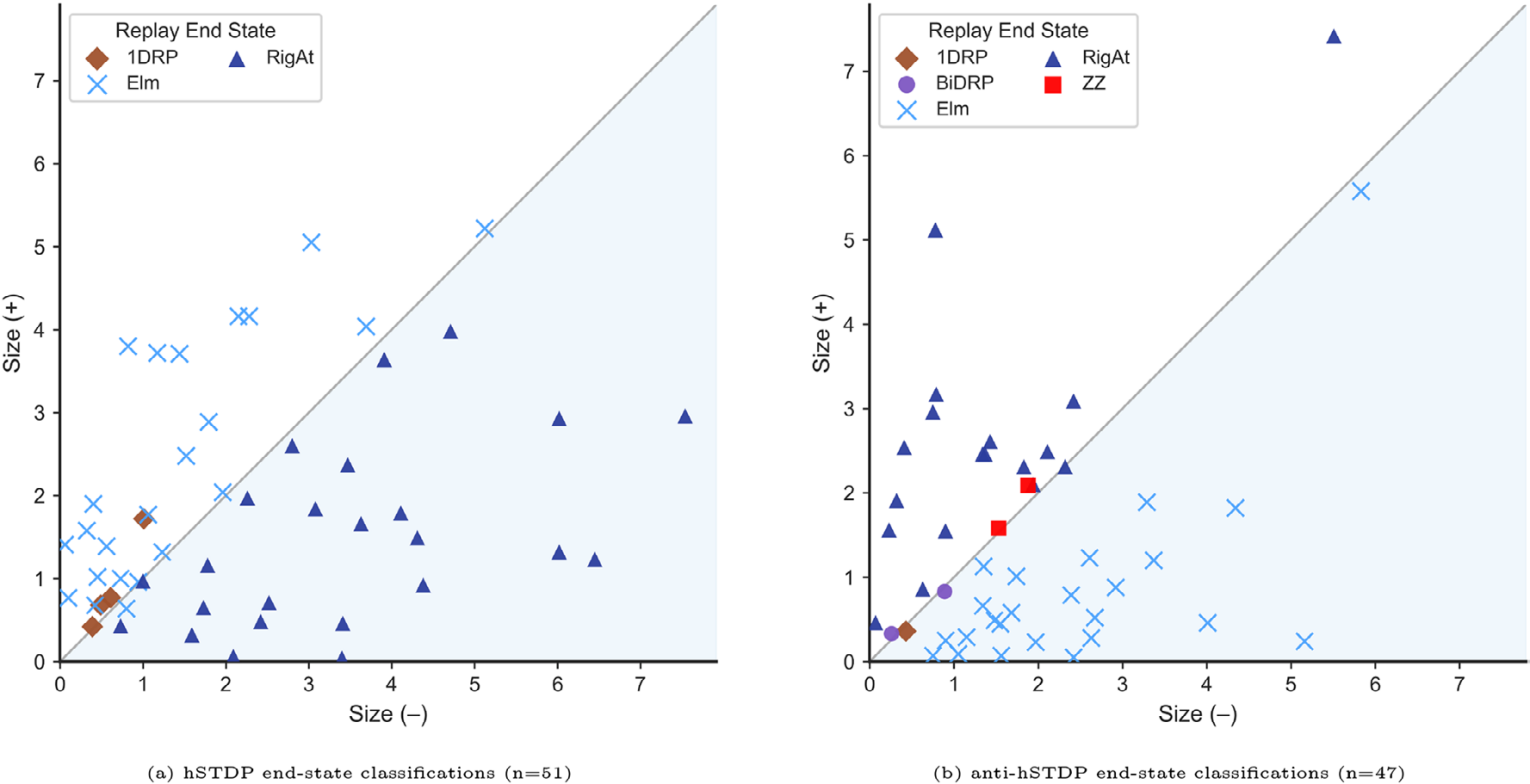
Replay progression under Plasticity Kernel Components (PKCs) of uneven size ($\tau_{+}\neq \tau_{-}$, $|A_{+}|\;\neq \;|A_{-}|$, and $\mathrm{sign}\left(A_{+}\right)\neq \mathrm{sign}\left(A_{-}\right)$). *Axes:* X‐axis shows the size of the negative‐timing PKC (${\text{Size}}_{-}$, see Section 2) in nS ms. Y‐axis shows the size of the positive‐timing PKC (${\text{Size}}_{+}$, see Section 2) in nS ms. *Legend:* Dark blue triangle = Rigid Attractor (RigAt), light blue “x” = Eliminator (Elm.), purple circle = Bi‐directional Replayer (BiDRP), red square = Zigzagger (ZZ), brown diamond = 1D Replayer. *Symmetry note:* The plot is symmetrically shaded—upper half corresponds to ${\text{Size}}_{+}>{\text{Size}}_{-}$ (PT‐dominated), and lower half to ${\text{Size}}_{-}>{\text{Size}}_{+}$ (NT‐dominated).

Next, we examined scenarios with asymmetric STDP kernels in which the plasticity components were uneven in size ($|A_{+}|\;\neq \;|A_{-}|$, $\tau_{+}\neq \tau_{-}$), to assess their impact on replay progression. We found that when the positive PKC component (A>0) was larger than the negative one, replays resembled st‐LTP‐like behavior, often leading to saturated activity patterns such as Rigid Attractors or Zigzag dynamics. In contrast, when the negative component was larger, simulations more closely resembled st‐LTD simulations, inducing rapid replay elimination (Figure 7). Thus, uneven kernel components yielded results similar to our symmetric kernel analyses.

### 3.6 Replay Speed Changes Are Driven by the Negative‐Timing Plasticity Components

Our observations regarding replay acceleration under hSTDP (See Section 3.3) prompted us to explore the impact of different plasticity kernels on replay speed. To assess this, we systematically activated specific Plasticity Kernel Components (PKCs) and measured changes in replay speed over time (See Section 2). This analysis was performed both for paired PKC activations—representing canonical STDP rules—and for isolated PKC components.

**FIGURE 8:**
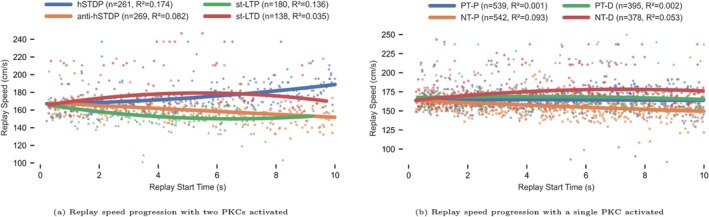
Effect of plasticity kernel activation on replay speed over time. X‐axis: Replay start time from simulation start (seconds). Y‐axis: Replay speed (cm/s). Each marker represents a replay event (n = number of observations). Solid lines show quadratic fit curves, with shaded areas indicating 95% confidence intervals. (a) Simulations with two PKCs activated (one for positive‐timing (PT) and one for negative‐timing (NT) components). (b) Simulations with a single PKC activated.

In the paired PKC experiments (Figure 8a), each rule produced a distinct pattern of replay speed modulation. hSTDP led to a gradual acceleration of replay speed during the early stages of the simulation, until the network transitioned into a Rigid Attractor or an Eliminator. In contrast, anti‐hSTDP initially caused a progressive deceleration of replay speed, which eventually stabilized. Simulations using st‐LTP also showed a consistent slowing of replay speed before transitioning to a Rigid Attractor state. Conversely, st‐LTD initially increased replay speed before transitioning to an Eliminator state.

To understand the impact of individual PKCs on replay speed, we conducted simulations in which only one PKC was active. To delay the onset of replay saturation or elimination and allow for extended observation, we used small kernel amplitudes and time constants ($\tau_{+/-}=10$ ms, $|A_{+/-}|\;=0.14$ nS) for all single PKC simulations.

When comparing PT‐P and NT‐P components, we found that both generated a high number of replays—569 and 626 respectively across 50 simulations each (Figure 8b). Replay speed under PT‐P initially increased but later slowed, while NT‐P showed a steady decline in speed throughout the simulation.

A similar pattern emerged for PT‐D and NT‐D. PT‐D produced a total of 359 replays and consistently slowed replay speed over time. NT‐D, on the other hand, exhibited an initial acceleration phase that lasted for approximately 10 s, followed by deceleration (Figure 8b). Although both of these depression‐driven PKCs contributed fewer total replays compared to their potentiation‐driven counterparts, they revealed complementary dynamics in speed modulation.

#### 3.6.1 Key Observations

Since all kernels with NT‐P active demonstrated replay slowdown (Figure 8a,b), we concluded that when a kernel with two PKCs of equal size is activated, NT‐P is crucial for replay deceleration. Similarly, all kernels with NT‐D active resulted in acceleration. We therefore concluded that NT‐D is crucial for replay acceleration. In addition, we attribute the switch from acceleration to deceleration when only PT‐P was active (Figure 8b) to the fact that under a PT‐P‐only kernel, the network continued to generate replays in both directions; thus, the impact on synaptic weights as the simulation progressed was similar to the impact of an st‐LTP kernel as the simulation progressed. We expect that the observed effects of the NT‐P and NT‐D components arise from the interaction between the activated subpopulation of PCs, firing rate adaptation (shown by Ecker et al. (2022) to be essential for replay generation), and the inhibitory BCs population. The potentiation of retrograde PCs' synapses works to counter the impact of the firing rate adaptation that pushes activity further along the track, thus prolonging the activity of PC subpopulations at the expense of downstream PCs that are suppressed by the inhibitory BCs. In other words, strong retrograde synapses help maintain the activity state within the localized group of PCs, effectively slowing down the motion of the attractor.

## 4 Discussion

### 4.1 Overview

We investigated how distinct synaptic plasticity rules applied during offline states modulate hippocampal replay dynamics. Using and replicating the model of Ecker et al. (2022), we confirmed that bidirectional replay can be generated with a symmetric st‐LTP rule during learning. Such a plasticity rule provides bidirectional strengthening of synapses, thus promoting the formation of associations that propel network dynamics both forward and backward relative to the temporal order of experience. This plasticity rule has gained recent traction following experimental evidence that it describes plasticity at the CA3‐CA3 and CA3‐CA1 synapses (Mishra et al. 2016; Falcón‐Moya et al. 2020) and its demonstrated capacity to promote bi‐directional replay (Ecker et al. 2022; Haga and Fukai 2018) in neuronal network models. A similar learning rule was also recently suggested for inhibitory synapses (Liao et al. 2024) to explain the selective recruitment of cue‐associated PCs into hippocampal replay ensembles. Nevertheless, it is intuitive that this learning rule cannot be the final word on synaptic plasticity mechanisms, because it exclusively potentiates synapses. Some form of synaptic weakening, either concurrently or subsequently, would appear needed to balance the potentiation and prevent the corruption of memory traces. Alternately, plasticity would need to be paused, as in the Ecker et al. (2022) study. We confirmed this intuition by observing the impact of the st‐LTP rule during the offline state, where the replays occur. As predicted, such a rule led to the saturation of synaptic connectivity in the network, followed by rigid or epileptic‐like activity.

It has been proposed that offline states improve the network's propensity for, and robustness of, memory recall by introducing a net weakening of synaptic connections during these states (Tononi and Cirelli 2014; Nere et al. 2013). We therefore considered the effects of a st‐LTD rule, similar to ones that have been proposed for the dorsomedial striatum (Perez et al. 2022) and for unclustered dendritic spines of CA1 neurons (Weber et al. 2016; Tazerart et al. 2020), that could produce uniform synaptic weakening during offline replay. This plasticity rule, unsurprisingly, led to the rapid elimination of all replay activities and elimination of the learned memory trace. This observation argues for a mechanism that allows for some reprieve from synaptic depression during offline states for at least a subset of connections, as also noted by Nere et al. (2013). They considered an offline plasticity rule where all synapses were downscaled except for those that participate in replays. It may be that the brain has evolved to implement such complex synapse‐specific learning rules through selective expressions of different proteins (Diering 2023) and/or compensatory circuit motifs (Karaba et al. 2024).

For simplicity, we next considered the standard asymmetric Hebbian STDP (hSTDP) rule that balances LTP and LTD. Though questions have been raised about the biological feasibility of this learning rule (Lisman and Spruston 2010; Shouval et al. 2010), it remains popular at least in part due to its computational benefits (Yang and Doiron 2026; Bayati et al. 2015; González et al. 2020). As we had expected (e.g., Haga and Fukai (2018), Ecker et al. (2022)), this rule introduced a pronounced directional bias in replays: forward replays reinforced the synaptic pathways that favored their propagation, while weakening retrograde connections, leading to progressive acceleration of replay. This was ultimately followed by the degradation of the memory trace through “decoupling through synchrony,” as described by Lubenov and Siapas (2008). In this mechanism, the conduction delays among the PCs lead to acausal (i.e., negative timing [NT]) firing during overly‐synchronized network population bursts, leading to LTD which destabilizes the network's synaptic structure. Consistent with this explanation, in simulations without conduction delays, we observed a marked slowdown in replay degradation.

### 4.2 Benefits of Anti‐Hebbian Plasticity

Our main finding is that, in contrast with the standard Hebbian STDP rule, a noncanonical anti‐Hebbian STDP (anti‐hSTDP) rule preserved bidirectional replay by weakening synapses consistent with the current direction of propagation while concurrently strengthening retrograde connections. This created a balancing dynamic that induced periodic switching of replay direction and stabilized the replay propagation speed. Unlike a recent model that used a strong lasting adaptation current to produce alternations between forward and reverse replay (Mallory et al. 2025), our model incorporated a relatively weaker spike‐frequency adaptation that promotes replay propagation (Ecker et al. 2022; Azizi et al. 2013), with the switches between forward and reverse replay occurring spontaneously under the influence of ongoing plasticity. Furthermore, replays under the anti‐hSTDP rule covered a greater portion of the track, as events that spontaneously started at one location tended to propagate all the way to the end of the track, unlike under different plasticity rules. This was likely due to the inherent balance provided by this learning rule, which weakens the initially stronger connections at track ends, but strengthens them in the opposite direction, thus increasing the chances of complete propagation, as well as of the emergence of replay in the opposite direction near the track ends. Component‐level analysis revealed that negative timing potentiation (NT‐P) was the dominant factor in slowing replay, while negative timing depression (NT‐D) drove acceleration. This is consistent with the viewpoint that replay speed is largely regulated by retrograde synapses, the strengthening (resp., weakening) of which results in deceleration (resp., acceleration). Interestingly, our findings also provide a mechanistic explanation for recent empirical observations by Berners‐Lee et al. (2022), who noted a correlation between progressive learning time and slower replays. They attributed this deceleration to the integration of more PCs into the replay process, which is not inconsistent with our results. However, we showed that anti‐hSTDP can also slow replay through the strengthening of retrograde connections, by counterbalancing the forward propulsion of the experienced trajectory, thus preserving the (temporary) stabilization of the (unstable) attractors that compose a learned (or generalized) trajectory.

While it remains unknown whether anti‐hSTDP may be in effect at hippocampal excitatory synapses, such a learning rule has been reported for corticostriatal projections (Schulz 2010; Morera‐Herreras et al. 2019) and for inhibitory synapses in the neocortex (Debanne and Inglebert 2023; Holmgren and Zilberter 2001). Recently, a striatal model by Vignoud et al. (2024) implemented an anti‐hSTDP rule, similar to ours, but under a very different network architecture, in the context of striatal sequence learning by a downstream network; this study found that anti‐hSTDP provided distinct advantages in sequence learning over hSTDP by increasing accuracy and allowing for better discrimination of partially overlapping sequences. As noted by these authors, the capability of anti‐Hebbian STDP to depress associated synapses “could naturally endow the system with the patience necessary to listen to full sequences and identify specific ones,” making this learning rule a natural one for self‐organized sequence learning.

### 4.3 Potential Mechanisms for State‐Dependent Plasticity Rules

Our study is grounded in the two‐stage model of memory formation in which neuronal activity and plasticity rules vary between online and offline states. During the online phase, repeated cycles of place‐cell firing during theta oscillations promote the initial formation of a memory trace (O'Neill et al. 2008; Skaggs et al. 1996). This is followed by the offline phase which is characterized by internally generated network firing that produces spontaneous replays during SWRs (Buzsáki 1989). Although we did not explicitly distinguish between immobile rest and sleep, given their neurophysiological similarities in the hippocampus (Findlay et al. 2025), we acknowledge that plasticity rules may further differ between these states (Miyawaki and Diba 2016; Giri et al. 2024). Furthermore, while our two‐state paradigm does not simulate the interleaved theta and replay sequences typical of pauses in exploration, we anticipate that the fundamental principles of replay modulation uncovered here remain applicable to those conditions.

What is the possible mechanism for a shift from st‐LTP during online learning to balanced plasticity rules, such as anti‐hSTDP, during offline states? The most likely mediators are neuromodulators, such as acetylcholine (ACh), dopamine (DA), and serotonin (5‐HT). The studies of Sugisaki et al. (2011) and Zhang et al. (2009) have shown that bath concentrations of ACh directly influence STDP: high ACh yields st‐LTP, while low ACh drives a shift toward asymmetric hSTDP. Since the hippocampus receives greater ACh from the medial septum during active exploration (Puhl et al. 2023), it is plausible that transitions from active exploration to immobility and rest would yield changes in hippocampal ACh that modify the operant plasticity rule. Other studies suggest that DA can also modify the plasticity rule by promoting st‐LTP (Wert‐Carvajal et al. 2022; Zhang et al. 2009), whereas serotonin can do so by selectively enhancing st‐LTD (Wert‐Carvajal et al. 2022; Hagena and Manahan‐Vaughan 2017). In light of these studies, it seems safe to assume that different plasticity rules are in effect under different brain states and change dynamically, from novelty to familiarity and from learning to rest and sleep. These rules may further differ between brain regions—even between different hippocampal subregions, such as CA3‐CA3 and CA3‐CA1 synapses (Liu et al. 2023; Cheng 2013)—potentially leading to differences in replay patterns across different regions.

### 4.4 Generality of Findings and Implications for Artificial Learning Systems

What role do the details of neuronal modeling play in the qualitative theory that emerges from these results? The perturbation experiments that tested and confirmed our conjectures regarding mechanism—including the systematic manipulation of plasticity components to isolate their role in system dynamics (Figure 8), and the systematic manipulation of synaptic delays to isolate the role of Lubenov and Siapas' synchrony by decoupling theory (Figure 5c,d)—did not depend on the details of single neuronal modeling. This lends some support to our qualitative hypothesis that these are generic features of recurrent neuronal networks that learn sequences via local synaptic plasticity mechanisms. Taken together, our findings offer a mechanistic framework linking synaptic plasticity rules to emergent replay behavior and provide insights into how offline synaptic dynamics may preserve, corrupt, and generalize memory traces (Wikenheiser and Redish 2013) during consolidation.

Our study also has implications for artificial neural networks that embody hippocampus‐inspired online learning. In the two‐stage model of hippocampal‐dependent memory, the hippocampus rapidly learns experienced patterns and uses replays to update synaptic weights in other brain structures. These models balance the requirement to rapidly learn relatively coarse multimodal patterns with the need to preserve the relatively finer structure of semantic knowledge (Marr 1971; McClelland et al. 1995; Buzsáki 1989; Diekelmann and Born 2010; Klinzing et al. 2019; Levy 1989). Levy in particular highlighted the sequential nature of such patterns in the context of a series of neurobiological considerations to support his proposal that sequence prediction unifies the computational roles of the hippocampus (Levy 1989, 1996; Levenstein et al. 2024).

The two‐stage model is increasingly adopted in artificial intelligence, which currently faces limitations in real‐time knowledge updating due to the phenomenon of new memories overwriting old memories, referred to as catastrophic forgetting (French 1999; Hasselmo 2017). Replay has enhanced performance in artificial learning systems in supervised, unsupervised, and reinforcement learning paradigms (González et al. 2020; Hayes et al. 2021; Tadros et al. 2022). Moreover, the generative nature of replay, which can produce a complete memory trace on demand from a cue and even generalize memory traces by recombination of existing memory traces (Ólafsdóttir et al. 2018), is reminiscent of generative AI models that achieve generalization by seeding (“prompting”) activity in memory systems that learn by adjusting node‐to‐node excitation parameters in directions determined by the goal of predicting input streams (Rolls 2024; Stoianov et al. 2022). Our work provides a biologically compatible model for the rapid, coarse‐learning components of the two‐stage model that features intrinsic and ethnologically persuasive generalization capacities. The results described here regarding the relative roles of plasticity rules on the degradation and preservation of memory traces are an exploration and reminder of problems biology must solve. They thus may provide inspiration for parallel challenges in artificial neuronal network research, including the transfer of memory traces, designed mechanisms for memory degradation, efficient synaptic coding, and the value and importance of these features for data‐efficient coarse‐to‐fine learning.

## Funding

This work was supported by the National Institute of Mental Health (R01MH139216) and the National Science Foundation (2423995, PHY‐230195).

## Conflicts of Interest

The authors declare no conflicts of interest.

## Data Availability

The data that support the findings of this study are available from the corresponding author upon reasonable request.

## Endnotes

^1^

https://www.microsoft.com/en‐us/sql‐server/sql‐server‐2022.

^2^

https://www.microsoft.com/en‐us/power‐platform/products/power‐bi/.

## Appendix A

### Data, Code, and Reproducibility

The experimental data generated and analyzed during this study are available from the corresponding author upon reasonable request.

The source code used to generate spike trains, train the network using STDP, and run the replay simulations is publicly available at:

https://github.com/sliorbar/ca3net.

Interactive visualizations and analysis dashboards developed for this study using Microsoft Power BI can be downloaded from:

https://drive.google.com/drive/folders/1PnCEREBjL5KjsZbDrLWq8RMfR2pJiFe1.

#### Hardware

All simulations were executed on a workstation running Windows 11 equipped with 48 GB RAM, an Intel Core i7 gen 8 CPU, and a 1 TB solid‐state drive.

#### Software

Model development and simulation code were written in Python 3.12 and developed using Visual Studio Code. Data storage and querying were performed using Microsoft SQL Server 2022. Statistical analysis and exploratory data analysis were conducted in Jupyter notebooks using Python 3.12. Visualization and interactive dashboards were created using Microsoft Power BI.
